# In steroid-resistant nephrotic syndrome that meets the strict definition, monogenic variants are less common than expected

**DOI:** 10.1007/s00467-024-06468-5

**Published:** 2024-08-02

**Authors:** Yuta Ichikawa, Nana Sakakibara, China Nagano, Yuta Inoki, Yu Tanaka, Chika Ueda, Hideaki Kitakado, Atsushi Kondo, Shingo Ishimori, Tomoko Horinouchi, Kazumoto Iijima, Kandai Nozu

**Affiliations:** 1 Department of Pediatrics, Kobe University Graduate School of Medicine, 7-5-1 Kusunoki-Cho, Chuo-Ku, Kobe, 650-0017 Japan; 2 Hyogo Prefectural Kobe Children’s Hospital, Kobe, Japan; 3 Department of Advanced Pediatric Medicine, Kobe University Graduate School of Medicine, Kobe, Japan

**Keywords:** Steroid-resistant nephrotic syndrome, Monogenic variants, Edema, Acute kidney injury associated with nephrotic syndrome, Complete remission

## Abstract

**Background:**

In patients with steroid-resistant nephrotic syndrome (SRNS), the presence of monogenic variants influences therapeutic strategies. Large cohort studies reported the detection of monogenic variants in approximately 30% of patients with SRNS. However, these cohorts included many patients, such as those with symptomatic proteinuria, who did not meet the strict diagnostic criteria for pediatric nephrotic syndrome (NS). Therefore, we investigated the proportion of causative monogenic variants detected in patients who strictly met the diagnostic criteria of SRNS and explored their clinical characteristics.

**Methods:**

We examined pediatric SRNS cases with genetic analysis conducted in our hospital. Cases satisfying all of the following criteria were included: (1) age at onset 1–18 years, (2) serum albumin at onset ≤ 2.5 g/dl, (3) persistent heavy proteinuria, and (4) no complete remission after 4 weeks of steroid monotherapy.

**Results:**

The proportion of detected monogenic variants was 12% (22/185) among all patients. The proportion was only 7% (9/129) in patients with edema at disease onset compared with 38% (9/24) in those without (*p* < 0.0001). Monogenic variants were rare in patients with acute kidney injury associated with NS (1% (1/11)) or a history of complete remission (4% (2/51)).

**Conclusions:**

Our study revealed a monogenic cause in 12% of individuals with strictly defined SRNS, a much smaller proportion than previously reported. The presence or absence of edema at the onset was an important factor to distinguish SRNS with monogenic cause from SRNS without. Our results provide further evidence of the SRNS types attributable to monogenic causes.

**Graphical abstract:**

**Figure:**
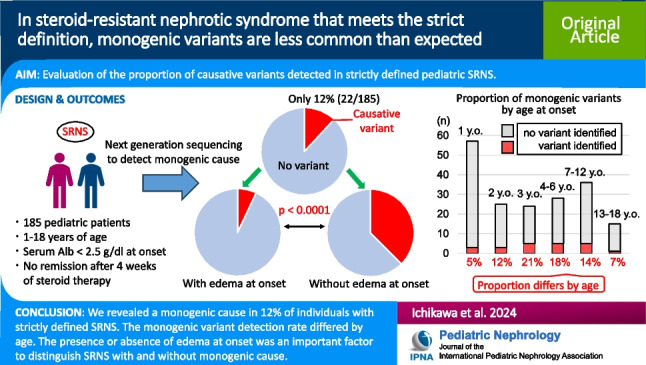
A higher resolution version of the Graphical abstract is available as Supplementary information

**Supplementary Information:**

The online version contains supplementary material available at 10.1007/s00467-024-06468-5.

## Introduction

Nephrotic syndrome (NS) is an umbrella term for glomerular filtration barrier dysfunction resulting in generalized edema because of heavy proteinuria and hypoalbuminemia. Per year, 2.0–6.5 per 100,000 children develop this syndrome [1, 2]. Approximately 90% of pediatric NS is categorized as idiopathic nephrotic syndrome (INS) [3], and 10–15% of these are steroid-resistant nephrotic syndrome (SRNS) without complete remission after 4–6 weeks of steroid therapy (60 mg/m^2^ or 2 mg/kg) [4]. In SRNS with immunological causes, combination therapy of steroid pulse therapy, cyclosporine A, and tacrolimus has shown favorable outcomes, with an efficacy rate of more than 80% [5, 6]. And the combination with rituximab has also been suggested in recent years to be effective [7, 8]. However, in SRNS associated with a monogenic variant, immunosuppressive therapy is ineffective in almost all cases [9, 10]. Thus, it is important to distinguish monogenic from immunological SRNS, but this often proves difficult in clinical practice. Genetic analysis is essential to decide the treatment strategy [4], but not all patients with SRNS can immediately undergo genetic analyses.

Based on analyses of large cohorts in Japan and other countries, genetic etiologies can be identified in about 30% of patients with SRNS [11–14]. However, these cohorts included cases that did not meet the International Study of Kidney Disease in Children (ISKDC) criteria of INS [15] and included asymptomatic proteinuria without nephrotic range hypoalbuminemia, familial focal segmental glomerulosclerosis (FSGS), congenital NS (onset before 3 months), and infantile NS (onset before 1 year). From a clinical perspective, monogenic SRNS represents less than 30% of all cases with SRNS, but surprisingly, the proportion of causative variants detected in strictly defined pediatric SRNS remains unclear.

In this study, we investigated the proportion of causative monogenic variants detected in properly diagnosed SRNS and what clinical manifestations are associated with monogenic SRNS.

## Materials and methods

### Patients

This study included pediatric patients diagnosed with SRNS between 1 and 18 years of age who underwent comprehensive gene screening between March 2016 and October 2022. Patients screened before December 2018 have already been reported by our group in a study about comprehensive genetic diagnosis of patients with severe proteinuria [11]. Children with congenital or infantile NS were excluded from the current study. Patients with serum albumin > 2.5 g/dl at onset or unknown serum albumin levels at onset were also excluded (Online Resource 1).

### Definitions

In this study, SRNS was redefined as a serum albumin concentration ≤ 2.5 g/dl at onset, persistent heavy proteinuria (> 40 mg/h/m^2^ in nocturnal urine or urine protein/creatinine ratio ≥ 2.0 g/gCr in morning urine) and no complete remission after 4 weeks of treatment with 60 mg/m^2^/day of prednisolone, according to the ISKDC criteria [16, 17]. Family history was defined as the presence of any type of urine abnormality or kidney disease in the parents or siblings of the study participants. History of temporary dialysis due to acute kidney injury associated with NS (NS-AKI) was defined as a prior dialysis due to NS-AKI with subsequent recovery of kidney function leading to weaning from dialysis. Chronic kidney disease (CKD) stage 5 was defined as a progressed stage of CKD with kidney replacement therapy (hemodialysis, peritoneal dialysis, or kidney transplantation). Complete remission was defined as dipstick-negative protein in morning urine or a morning urine protein-to-creatinine ratio < 0.2 g/gCr for 3 consecutive days.

### Assessment of clinical findings

The following items were extracted from questionnaires obtained from the local doctors of the patients: (1) age at onset, (2) age at genetic analysis, (3) sex, (4) edema at onset, (5) family history, (6) extrarenal complications, (7) history of temporary dialysis due to NS-AKI prior to the genetic analysis, (8) CKD stage 5 prior to the genetic analysis, (9) history of complete remission prior to the genetic analysis, and (10) initial histopathological diagnosis.

### Genetic analysis

Genetic analysis was performed as previously reported [18]. In brief, genomic DNA was isolated from peripheral blood leukocytes obtained from the participants and their families. For the custom next-generation sequencing panel targeting podocyte-related genes (Online Resource 2), samples were prepared using the Haloplex or Sure Select target enrichment system kit (Agilent Technologies, Santa Clara, CA, USA), in accordance with the manufacturer’s instructions. All indexed DNA samples were amplified by polymerase chain reaction and sequenced using the MiSeq platform (Illumina, San Diego, CA, USA).

We performed custom array comparative genomic hybridization (aCGH) as previously reported [19] for one case. We conducted pair analysis using the SureCall application in this patient and suspected that this patient had a large heterozygous deletion including the *NUP85* gene (chromosome 17, q25.1 partial deletion).

We used the computational prediction software SIFT (https://research.a-star.edu.sg/tag/sift/), PolyPhen-2 (http://genetics.bwh.harvard.edu/pph2/), Mutation Taster (https://www.mutationtaster.org/), and CADD (https://cadd.gs.washington.edu/snv) to classify variants as pathogenic, likely pathogenic, or of uncertain significance, according to the guidelines of the American College of Medical Genetics and Genomics [20].

### Statistical analysis

Results are presented as median and interquartile range (IQR). The chi-squared test or Fisher’s exact test was used to compare variables between each group. Wilcoxon’s rank-sum test was used to compare median differences in age of onset between each experimental group. Statistical analysis was performed using standard statistical software (JMP version 14 for Windows; SAS Institute, Cary, NC, USA). In all tests, *p* < 0.05 was considered statistically significant.

## Results

In total, 185 patients met the inclusion criteria (Online Resource 1). The clinical characteristics of the patients are shown in Online Resource 3. The median age at onset was 3 years (IQR, 2.0–10.5 years), and the median age at the time of genetic analysis was 5 years (IQR, 1.0–7.0 years). The male to female ratio was 10.0:7.6. Most patients, including all affected by monogenic disorders, were treated with at least one additional immunosuppressant.

**Fig. 1:**
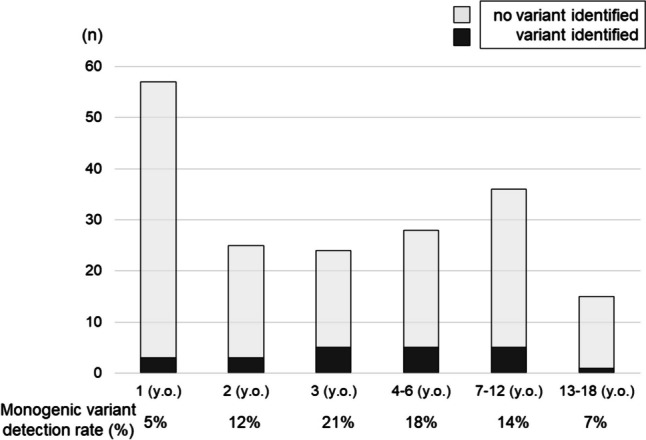
Proportion of monogenic variants by age at onset. Black and gray bars show whether causative variants were detected or not, respectively. The rates were lower at 1 year (5%) and 2 years (12%) of age but peaked at 3 years of age (21%). The rate was again lower for those over 13 years of age, but the rates in the age groups 4–6 and 7–12 years were relatively high. Abbreviation: y.o., years old

Causative monogenic variants were identified in 12% (22 of 185) patients; *WT1* gene variants had the highest frequency with six cases (Table 1). The genotype details of the 22 cases in which disease-causing variants were identified are shown in Online Resource 4, and the details of the aCGH results in the patient with suspected large deletion in *NUP85* are shown in Online Resource 5. The proportions of detected causative variants by age group (Fig. 1) were 5% at 1 year of age, 12% at 2 years of age, 21% at 3 years of age, 18% at 4–6 years of age, 14% at 7–12 years of age, and 7% at 13–18 years of age.

**Table 1:** Genes with disease-causing variants in 185 patients with steroid-resistant nephrotic syndrome

Causative gene	*n*
*WT1*	6
*TRPC6*	3
*COL4A4*	1
*NPHS1*	2
*PLCE1*	2
*SMARCAL1*	2
*ACTN4*	1
*ARHGAP24*	1
*INF2*	1
*LAMB2*	1
*NUP85*	1
*NUP93*	1
Causative variant not detected	163

The clinical phenotypes of patients with and without variants are compared in Table 2. The groups with and without identified variants significantly differed in sex (*p* = 0.0455), presence of edema at onset (*p* < 0.0001), and history of complete remission (*p* = 0.01). The proportion of monogenic variants was only 7% (9 of 129) in patients with edema at disease onset but 38% (9 of 24) in those without edema at onset. However, no significant difference in the age at onset, family history, extrarenal complications, history of temporary dialysis due to NS-AKI, history of CKD stage 5, and initial histopathological diagnosis was found between the group with identified variants and that without. Notably, monogenic causes were rare in patients with NS-AKI (1% (1 of 11)) or those with a history of complete remission (4% (2 of 51)).

**Table 2:** Comparison of clinical phenotypes between patients with and without variants in the analyzed genes

Clinical phenotype	Total	Patients with variants	Patients without variants	*p* value
Age at onset (years)^a^	3 (2.0–10.5)	4 (2.0–7.0)	3 (1.0–7.0)	0.2948
Male sex, % (*n*/*N*)	57% (105/185)	36% (8/22)	60% (97/163)	0.0455
Absence of edema, % (*n*/*N*)	16% (24/153)	50% (9/18)	11% (15/135)	< 0.0001
Family history, % (*n*/*N*)	2% (3/185)	5% (1/22)	1% (2/163)	0.3175
Extrarenal complications, % (*n*/*N*)	17% (32/185)	23% (5/22)	17% (27/163)	0.5541
NS-AKI with temporary dialysis, % (*n*/*N*)	2% (4/185)	0% (0/22)	2% (4/163)	> 0.9999
CKD stage 5, % (*n*/*N*)	9% (17/185)	18% (4/22)	8% (13/163)	0.1250
Complete remission, % (*n*/*N*)	19% (35/185)	5% (1/22)	21% (34/163)	0.0100
Initial histopathologic diagnosis, % (*n*/*N*)				0.2258
FSGS	50% (76/153)	71% (12/17)	47% (64/136)	
MGA	41% (62/153)	24% (4/17)	43% (58/136)	
DMS	2% (3/153)	6% (1/17)	1% (2/136)	
DMP	8% (12/153)	0% (0/17)	9% (12/136)	

*CKD*, chronic kidney disease; *DMP*, diffuse mesangial proliferation; *DMS*, diffuse mesangial sclerosis; *FSGS*, focal segmental glomerular sclerosis; *MGA*, minor glomerular abnormalities; *NS-AKI*, acute kidney injury in nephrotic syndrome

^a^Median (interquartile range)

## Discussion

This study showed that monogenic variants in patients meeting the strict SRNS definition account for only 12%, i.e., they are less common than previously reported [11–14]. The results also demonstrated that the presence of edema is an important parameter to differentiate between SRNS types with a monogenic cause and those without.

A recent report from the UK identified causative variants in 10% of non-syndromic SRNS, excluding congenital and infantile NS [21]. Although their study did not examine serum albumin levels, the patient population in their study might be similar to ours, suggesting that our ratio of 12% is reasonable.

In a previous worldwide cohort, the proportion of families with detected monogenic cause at 1, 2, and 3 years of age remained almost unchanged at approximately 25% [12], while in the present study, the proportion according to age at onset was higher at 3 years of age. This difference may be related to urine screening of 3-year-olds during checkups in Japan. In other words, in Japan, asymptomatic proteinuria, which is the typical presentation of monogenic NS, is typically detected at 3 years of age. These cases are unlikely to have been identified without a urine screening. In addition, the proportion of causative variants detected at 1 year of age in our study was, surprisingly, 5%. This is possibly due to the high incidence of INS at 1 year of age. However, this result contrasts with the high probability of detection of monogenic variants in congenital and infantile NS. Moreover, as in our study, a certain number of cases with monogenic causes were found even beyond school age; it should be considered that monogenic SRNS is not limited to a specific age group.

The most important point of this study is that the detection of monogenic variants was low (7%) in typical cases with edema at onset but very high (38%) in atypical cases without edema at onset. Our group has previously reported that in a cohort of patients with severe proteinuria, significantly fewer patients with monogenic variants showed edema than those without [11], but it included several patients without hypoalbuminemia. The present study shows, for the first time, that in a cohort of only patients with hypoalbuminemia, those with monogenic variants had a significantly reduced frequency of edema.

In patients with congenital analbuminemia, edema is rare, possibly due to edema-preventive mechanisms, including mitigating effects on the oncotic gradient, a reduction in the hydrostatic blood pressure gradient, and a decrease in the capillary permeability of proteins [22]. Moreover, patients with congenital NS can often escape after some time an albumin-dependent status despite hypoalbuminemia [23]. Patients with monogenic variants are less likely to present with edema possibly because edema-compensatory mechanisms play a role in chronic or slowly progressive hypoalbuminemia.

In addition to edema, a significant sex difference was observed between the study groups. The significantly lower detection rate of gene abnormalities in males may be due to the higher incidence of INS in male patients. In addition, all six patients with *WT1* were female and this may reflect a strong bias, probably because male individuals with genital abnormalities were identified in infancy and therefore excluded from the study. The small sample size could have heightened these biases.

Furthermore, a significant difference was observed regarding a history of complete remission. Similar to the findings of a previous report [24], our results confirmed that monogenic variants were usually not identified in cases with a history of complete remission, except for two cases with remission after cyclosporine A administration (one case each with a *WT1* variant: Neph379 and a *COL4A4* variant: Neph573). It has been reported that some patients with NS associated with monogenic variants may respond to calcineurin inhibitors [25], but the two cases of Neph379 and Neph573 had a somewhat unusual clinical course and may have been accompanied by INS. In Neph379 (with the *WT1* variant), the urinary protein to creatinine ratio was approximately 20 g/gCr at the time of onset, and complete remission was achieved after administration of cyclosporine A, which continued for at least 1 year. Neph573 (with the *COL4A4* variant) also presented significantly heavy proteinuria (20 g/gCr for the urinary protein to creatinine ratio) that could not be solely explained by *COL4A4* abnormalities, suggesting a high probability of concomitant NS. However, the severity of NS in this patient may have been related to the *COL4A4* variant. In these two cases, although cyclosporin A could have contributed to achieving remission to some degree, it probably contributed more as an immunologic factor than as a genetic one.

Although no significant difference was observed in other factors, probably due to the small number of cases, some important findings should be highlighted. Monogenic variants were generally not identified in cases with NS-AKI that required temporary dialysis except for one case with a *COL4A4* variant (Neph573). As mentioned above, this case may have also presented additional complication due to concomitant immunologically induced NS. The reason why SRNS with monogenic variants is less likely to be accompanied by NS-AKI seems to be that NS develops slowly. In addition, the prevalence of monogenic variants was in this study not particularly high in patients with CKD stage 5. Based on these findings, some cases with CKD stage 5 include immunologically induced SRNS, providing the potential for improving kidney outcomes with more intensive immunosuppressive therapy, such as rituximab.

Regarding the initial histopathologic diagnosis, a high proportion (71%) of 246 monogenic variants was found to show FSGS in this study, similar to 63–74% reported in previous publications [11–14]. However, an initial histopathological diagnosis of minor glomerular abnormalities should not lead to the assumption of a non-monogenic cause because minor glomerular abnormalities may change to FSGS over the course of the disease. Especially in younger patients, the histology might be evaluated in the early stage of the disease.

This study has several limitations. First, not all pediatric patients diagnosed with SRNS underwent genetic analysis. The population referred to our hospital for genetic analysis might be biased towards complicated cases in which local doctors suspect monogenic causes. In practice, the proportion of monogenic causes seems to be much lower. Second, some of the cases without monogenic variants might include cases with genetic abnormalities that cannot be detected by our panels, such as deep intronic variants or genes with no established pathogenicity. Third, the timing of genetic analysis varied among patients; in some cases, genetic analysis was performed immediately after the diagnosis of SRNS, whereas in others, genetic analysis was performed after reaching CKD stage 5. Fourth, this study may be biased by the exclusion of congenital and infantile NS cases, both with a high rate of monogenic origin. Thus, the observed 12% should not be directly compared to the literature-based 30% [11–14]. However, both congenital and infantile NS cases often undergo genetic testing before steroid administration, and many of these patients had not received 4 weeks of steroid treatment, which was an inclusion criterion for this study. Therefore, we excluded patients under 1 year of age, which is one of the limitations of this study.

In the end, even in cases where monogenic variants were identified, most patients received additional immunosuppressive therapy (steroid pulse therapy, cyclophosphamide, rituximab, cyclosporine A, tacrolimus, mizoribine, or mycophenolate mofetil) until the results of the genetic analysis were known. This suggests that determining the presence or absence of genetic abnormalities from clinical history alone is challenging.

Although it is ideal for all patients with suspected SRNS to undergo genetic analysis promptly, cases with edema at disease onset should be more aggressively considered for additional immunosuppressive treatment. Conversely, in patients without edema, additional immunosuppressive treatment should be considered more carefully. We hope that our results may help pediatric nephrologists in the decision-making for SRNS therapy.

## Supplementary Information

Below is the link to the electronic supplementary material.

Graphical abstract (PPTX 230 KB)

ESM_1 (DOCX 119 KB)

ESM_2 (DOCX 30 KB)

ESM_3 (XLSX 28 KB)

ESM_4 (XLSX 17 KB)

ESM_5 (DOCX 231 KB)

## Data Availability

Data from this study can be obtained from the corresponding authors on reasonable request.
