# Supplementary material for: In steroid-resistant nephrotic syndrome that meets the strict definition, monogenic variants are less common than expected
Source: Pediatr Nephrol. 2024 Aug 2;39(12):3497–503. doi: 10.1007/s00467-024-06468-5 (PMC11511720; doi:10.1007/s00467-024-06468-5)
Supplement: Supplementary file 1 — Graphical abstract (PPTX 230 KB) [file 467_2024_6468_MOESM1_ESM.pptx]

## Slide 1
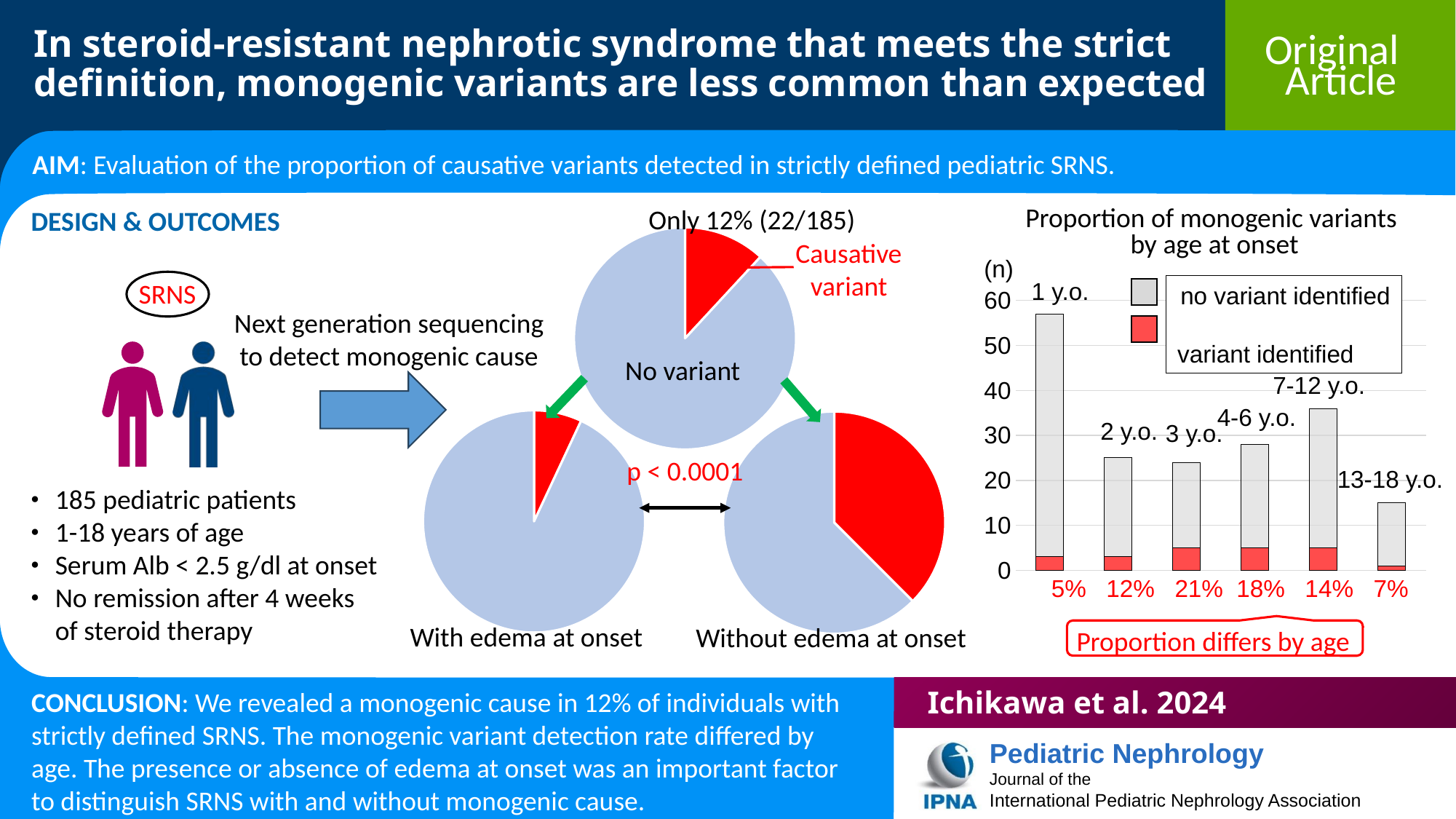

In steroid-resistant nephrotic syndrome that meets the strict definition, monogenic variants are less common than expected
AIM: Evaluation of the proportion of causative variants detected in strictly defined pediatric SRNS.
Only 12% (22/185)
DESIGN & OUTCOMES
Proportion of monogenic variants
by age at onset
### Chart
| Category | 売上高 |
|---|---|
| Causative variant | 22.0 |
| No variant | 163.0 |Causative
variant
(n)
1 y.o.
SRNS
no variant identified
variant identified
### Chart
| Category | | |
|---|---|---|
| 1 (y.o.) | 3.0 | 54.0 |
| 2 (y.o.) | 3.0 | 22.0 |
| 3 (y.o.) | 5.0 | 19.0 |
| 4-6 (y.o.) | 5.0 | 23.0 |
| 7-12 (y.o.) | 5.0 | 31.0 |
| 13-18 (y.o.) | 1.0 | 14.0 |Next generation sequencing
to detect monogenic cause
No variant
7-12 y.o.
4-6 y.o.
### Chart
| Category | 売上高 |
|---|---|
| Causative variant | 9.0 |
| No variant | 120.0 |
### Chart
| Category | 売上高 |
|---|---|
| Causative variant | 9.0 |
| No variant | 15.0 |2 y.o.
3 y.o.
p < 0.0001
13-18 y.o.
・185 pediatric patients
・1-18 years of age
・Serum Alb < 2.5 g/dl at onset
・No remission after 4 weeks
　of steroid therapy
5% 12% 21% 18% 14% 7%
With edema at onset
Without edema at onset
Proportion differs by age
Ichikawa et al. 2024
CONCLUSION: We revealed a monogenic cause in 12% of individuals with strictly defined SRNS. The monogenic variant detection rate differed by age. The presence or absence of edema at onset was an important factor to distinguish SRNS with and without monogenic cause.
