# Supplementary material for: In steroid-resistant nephrotic syndrome that meets the strict definition, monogenic variants are less common than expected
Source: Pediatr Nephrol. 2024 Aug 2;39(12):3497–503. doi: 10.1007/s00467-024-06468-5 (PMC11511720; doi:10.1007/s00467-024-06468-5)
Supplement: Supplementary file 2 — ESM_1 (DOCX 119 KB) [file 467_2024_6468_MOESM2_ESM.docx]

Supplementary data

**In steroid-resistant nephrotic syndrome that meets the strict definition, monogenic variants are less common than expected**

Yuta Ichikawa^1^, Nana Sakakibara^1^, Yuta Inoki^1^, Yu Tanaka^1^, Chika Ueda^1^, Hideaki Kitakado^1^, Atsushi Kondo^1^, China Nagano^1^, Tomoko Horinouchi^1^, Kazumoto Iijima^2,3^, Kandai Nozu^1^

1. Department of Pediatrics, Kobe University Graduate School of Medicine, Kobe, Japan.
2. Hyogo Prefectural Kobe Children's Hospital, Kobe, Japan.
3. Department of Advanced Pediatric Medicine, Kobe University Graduate School of Medicine, Kobe, Japan.

**Corresponding author**

Yuta Ichikawa MD

Department of Pediatrics, Kobe University Graduate School of Medicine, 7-5-1 Kusunoki-cho, Chuo-ku, Kobe 650-0017, Japan

Tel: +81-382-6090; Fax: +81-382-6099; E-mail: y0gobro@med.kobe-u.ac.jp


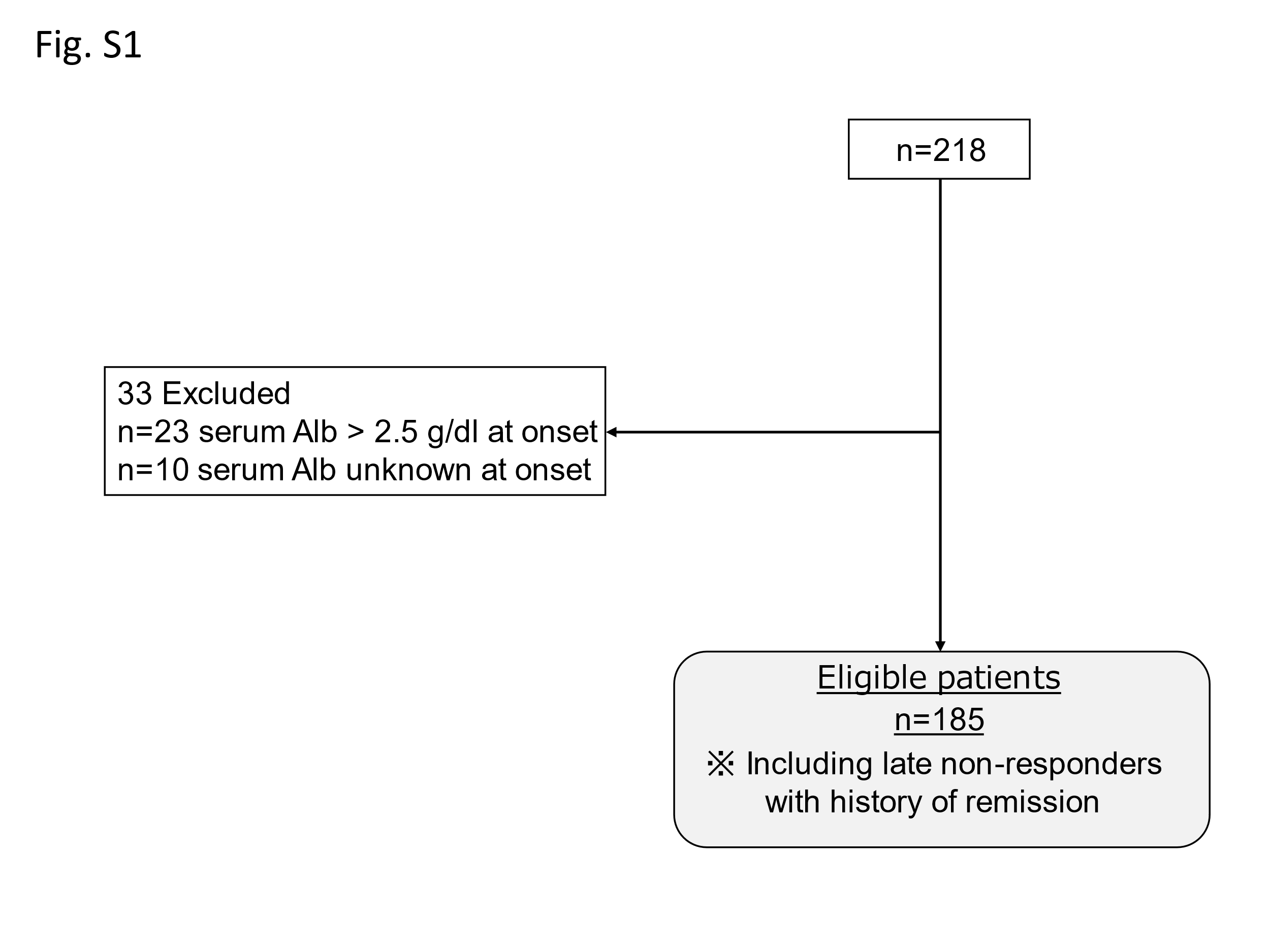
 **Fig. S1** This study included 218 pediatric patients diagnosed with SRNS between 1 and 18 years of age who underwent comprehensive gene screening between March 2016 and October 2022. We excluded 23 patients who started steroid therapy with serum Alb > 2.5 g/dl at onset and 10 patients with unknown serum Alb levels. Thus, data from 185 patients with a strict SRNS diagnosis were analyzed. Abbreviations: Alb, albumin; SRNS, steroid-resistant nephrotic syndrome
