# Supplementary material for: In steroid-resistant nephrotic syndrome that meets the strict definition, monogenic variants are less common than expected
Source: Pediatr Nephrol. 2024 Aug 2;39(12):3497–503. doi: 10.1007/s00467-024-06468-5 (PMC11511720; doi:10.1007/s00467-024-06468-5)
Supplement: Supplementary file 3 — ESM_2 (DOCX 30 KB) [file 467_2024_6468_MOESM3_ESM.docx]

Supplementary data

**In steroid-resistant nephrotic syndrome that meets the strict definition, monogenic variants are less common than expected**

Yuta Ichikawa^1^, Nana Sakakibara^1^, Yuta Inoki^1^, Yu Tanaka^1^, Chika Ueda^1^, Hideaki Kitakado^1^, Atsushi Kondo^1^, China Nagano^1^, Tomoko Horinouchi^1^, Kazumoto Iijima^2,3^, Kandai Nozu^1^

1. Department of Pediatrics, Kobe University Graduate School of Medicine, Kobe, Japan.
2. Hyogo Prefectural Kobe Children's Hospital, Kobe, Japan.
3. Department of Advanced Pediatric Medicine, Kobe University Graduate School of Medicine, Kobe, Japan.

**Corresponding author**

Yuta Ichikawa MD

Department of Pediatrics, Kobe University Graduate School of Medicine, 7-5-1 Kusunoki-cho, Chuo-ku, Kobe 650-0017, Japan

Tel: +81-382-6090; Fax: +81-382-6099; E-mail: y0gobro@med.kobe-u.ac.jp

**Table S1** List of the 68 podocyte-related genes included in the targeted sequencing analysis within a clinically approved gene panel test developed in our laboratory

| *ACTN4* | *COQ2* | *ITSN1* | *MAGI2* | *PAX2* | *TPRKB* |
| --- | --- | --- | --- | --- | --- |
| *ADCK4* | *COQ6* | *ITSN2* | *MYH9* | *PDSS2* | *TRIM8* |
| *ANKFY1* | *CRB2* | *KANK1* | *MYO1E* | *PLCE1* | *TRPC6* |
| *ANLN* | *CUBN* | *KANK2* | *NPHS1* | *PODXL* | *TTC21B* |
| *ARHGAP24* | *DLC1* | *KANK4* | *NPHS2* | *PRDM15* | *WDR4* |
| *ARHGDIA* | *EMP2* | *KIRREL1* | *NUP85* | *PTPRO* | *WDR73* |
| *AVIL* | *FAT1* | *LAGE3* | *NUP93* | *SCARB2* | *WT1* |
| *CD2AP* | *GAPVD1* | *LAMA5* | *NUP107* | *SGPL1* | *XPO5* |
| *CKD20* | *GON7* | *LAMB2* | *NUP133* | *SMARCAL1* |  |
| *COL4A3* | *INF2* | *LMNA* | *NUP160* | *TNS2* |  |
| *COL4A4* | *ITGA3* | *LMX1B* | *NUP205* | *TBC1D8B* |  |
| *COL4A5* | *ITGB4* | *MAFB* | *OSGEP* | *TP53RK* |  |
