# Supplementary material for: In steroid-resistant nephrotic syndrome that meets the strict definition, monogenic variants are less common than expected
Source: Pediatr Nephrol. 2024 Aug 2;39(12):3497–503. doi: 10.1007/s00467-024-06468-5 (PMC11511720; doi:10.1007/s00467-024-06468-5)
Supplement: Supplementary file 6 — ESM_5 (DOCX 231 KB) [file 467_2024_6468_MOESM6_ESM.docx]

Supplementary data

**In steroid-resistant nephrotic syndrome that meets the strict definition, monogenic variants are less common than expected**

Yuta Ichikawa^1^, Nana Sakakibara^1^, Yuta Inoki^1^, Yu Tanaka^1^, Chika Ueda^1^, Hideaki Kitakado^1^, Atsushi Kondo^1^, China Nagano^1^, Tomoko Horinouchi^1^, Kazumoto Iijima^2,3^, Kandai Nozu^1^

1. Department of Pediatrics, Kobe University Graduate School of Medicine, Kobe, Japan.
2. Hyogo Prefectural Kobe Children's Hospital, Kobe, Japan.
3. Department of Advanced Pediatric Medicine, Kobe University Graduate School of Medicine, Kobe, Japan.

**Corresponding author**

Yuta Ichikawa MD

Department of Pediatrics, Kobe University Graduate School of Medicine, 7-5-1 Kusunoki-cho, Chuo-ku, Kobe 650-0017, Japan

Tel: +81-382-6090; Fax: +81-382-6099; E-mail: y0gobro@med.kobe-u.ac.jp


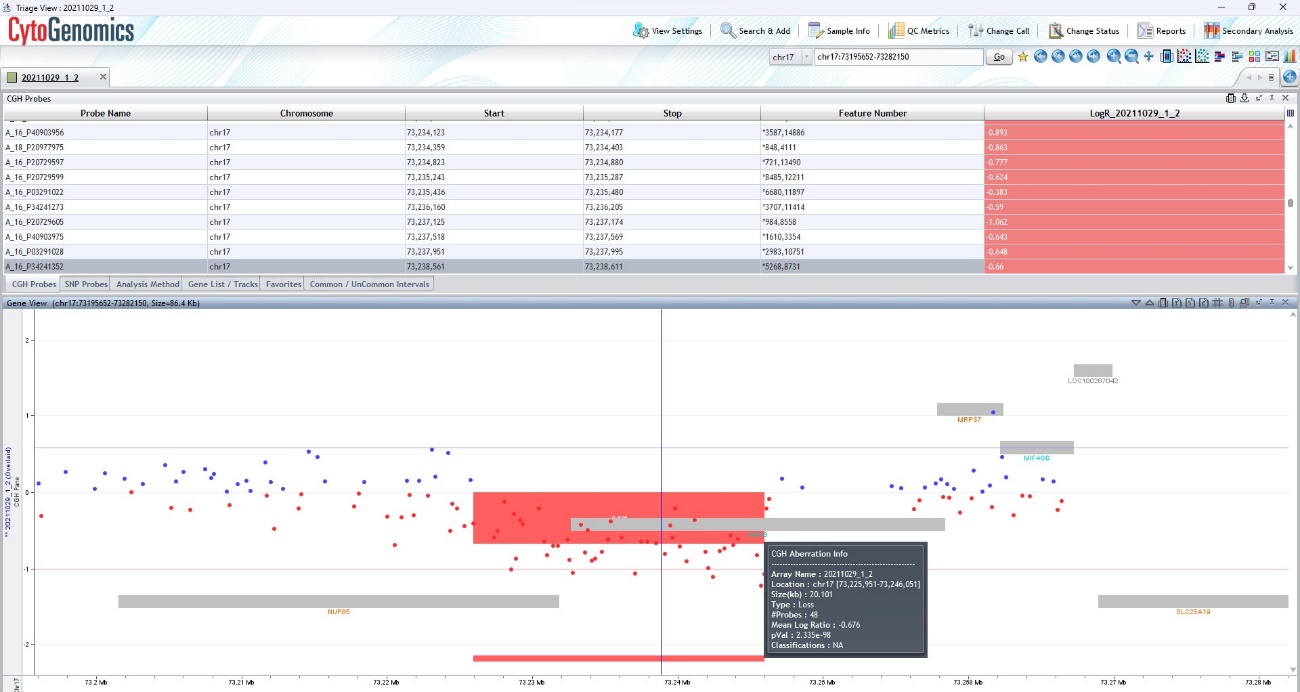
 **Fig. S2** Gene analysis for case Neph329. Targeted next-generation sequencing showed one novel heterozygous missense variant in *NUP85* [c.1379G>A, p.(Arg460Gln)]. We also conducted Sanger sequencing and detected the same mutation in both the patient and his father. When we conducted pair analysis using the SureCall application, a copy number variant of *NUP85* was suspected, so custom array comparative genomic hybridization was performed and detected that heterozygous chromosome 17 q25.1 partial deletion affected the *NUP85* gene region
